# The mechanism of pleural inflammation by long carbon nanotubes: interaction of long fibres with macrophages stimulates them to amplify pro-inflammatory responses in mesothelial cells

**DOI:** 10.1186/1743-8977-9-8

**Published:** 2012-04-03

**Authors:** Fiona A Murphy, Anja Schinwald, Craig A Poland, Ken Donaldson

**Affiliations:** 1 MRC/University of Edinburgh Centre for Inflammation Research, ELEGI Colt Laboratory, Queen's Medical Research Institute, 47 Little France Crescent, Edinburgh EH16 4TJ, UK; 2 Institute of Occupational Medicine, Research Avenue North, Riccarton, Edinburgh EH14 4AP, UK

**Keywords:** Carbon nanotubes, Pleura, Mesothelioma, Asbestos, Inflammation

## Abstract

Carbon nanotubes (CNT) are high aspect ratio nanoparticles with diameters in the nanometre range but lengths extending up to hundreds of microns. The structural similarities between CNT and asbestos have raised concern that they may pose a similar inhalation hazard. Recently CNT have been shown to elicit a length-dependent, asbestos-like inflammatory response in the pleural cavity of mice, where long fibres caused inflammation but short fibres did not. However the cellular mechanisms governing this response have yet to be elucidated. This study examined the in vitro effects of a range of CNT for their ability to stimulate the release of the acute phase cytokines; IL-1β, TNFα, IL-6 and the chemokine, IL-8 from both Met5a mesothelial cells and THP-1 macrophages. Results showed that direct exposure to CNT resulted in significant cytokine release from the macrophages but not mesothelial cells. This pro-inflammatory response was length dependent but modest and was shown to be a result of frustrated phagocytosis. Furthermore the indirect actions of the CNT were examined by treating the mesothelial cells with conditioned media from CNT-treated macrophages. This resulted in a dramatic amplification of the cytokine release from the mesothelial cells, a response which could be attenuated by inhibition of phagocytosis during the initial macrophage CNT treatments. We therefore hypothesise that long fibres elicit an inflammatory response in the pleural cavity via frustrated phagocytosis in pleural macrophages. The activated macrophages then stimulate an amplified pro-inflammatory cytokine response from the adjacent pleural mesothelial cells. This mechanism for producing a pro-inflammatory environment in the pleural space exposed to long CNT has implications for the general understanding of fibre-related pleural disease and design of safe nanofibres.

## Background

Carbon nanotubes (CNT) are high aspect ratio nanoparticles comprising single (SWCNT) or concentrically stacked multiwalled (MWCNT) graphene sheets rolled seamlessly into a cylinder. Their high aspect-ratio and novel properties make CNT a useful industrial material and has led to their incorporation into a wide variety of consumer products. However, as the applications of CNT continue to grow, so too does the potential for occupational inhalation exposure with obvious potential hazards for worker health [1]. The structural similarities between CNT and asbestos have raised particular concern regarding the potential pathogenicity of CNT in the lung and serosal cavities, specifically the pleural and peritoneal spaces, which are key target tissues for asbestos-related disease [2].

Carbon nanotubes have been found to cause a range of pathogenic effects, oxidative stress [3] inflammation and NLRP3 inflammasome activation [4], fibrosis [5,6] and genotoxicity [7].

The mesothelial lining of the pleural cavity has long been known to be particularly sensitive to asbestos exposure producing pleural effusion, pleural plaques and fibrosis [8]. Cancer arising in the mesothelial cells lining both the peritoneal and pleural cavities, mesothelioma, is a response almost unique to fibrous particles. The exact mechanisms leading to fibre-induced mesothelioma formation are unknown although fibre dimensions [9-11], biopersistence [12], the generation of reactive oxygen species (ROS) [13] and inflammation [14] have all been implicated. Due to its uniformly poor prognosis, mesothelioma is the disease of most concern when contemplating the potential toxicity of new high aspect ratio nanoparticles; pleural plaques and effusion are also a consequence of long fibre dose in the pleural space. The ability of fibres to induce an inflammatory response in the pleura has been considered to be a key mechanism in the production of mesothelioma and other pleural pathology[15,16]. A length dependent inflammatory response, similar to that seen with asbestos, has been reported for CNT and other high aspect ratio nanomaterials (HARN) in a number of studies using the peritoneal cavity as a model of mesothelium exposure [17,18] and more recently in a study conducted by the present authors investigating the response to CNT instilled into the pleural cavity [19]. The length-dependent response in the pleural cavity was characterised by an initial acute inflammatory reaction as indicated by an influx of granulocytes and an increase in protein concentration in the lavage fluid [19]. The length-dependent response to CNT in vivo was attributed to the fibre length-restricted clearance mechanisms from the pleural space through stomata in the parietal pleura leading to specific retention of long fibres while short fibres are efficiently cleared [19]. Nevertheless the detailed interactions between the CNT and pleural mesothelial cells and macrophages at these points of retention are unknown and are the focus of the present study.

Inhaled fibres that reach the pleural space will encounter mesothelial cells lining the pleural cavity and also resident pleural macrophages. Mesothelial cells, historically thought of as barrier cells that provide a lubricated surface that locks the parietal pleura to the visceral pleura allowing for lung movements, are now also known to play a prominent role in the initiation, perpetuation and resolution of inflammation in the pleural cavity [20]. During injury or infection, mesothelial cells can respond by producing a spectrum of pro- and anti-inflammatory cytokines and chemokines, growth factors, oxidants, extracellular matrix molecules and mediators of the complement cascade [20]. Macrophages are also present in the pleural space and can play a role in host defence and immuno-inflammatory responses [21]. The primary function of professional phagocytes such as pleural macrophages is to ingest foreign material that may pose a threat to the body and so pleural macrophages are likely to play a key role in the removal of inhaled particles and fibres that are retained in the pleural space. We therefore hypothesised that the response of the mesothelial cells and macrophages upon exposure to CNT or other fibres will be crucial in the pathogenesis of fibre-related pleural inflammation and therefore in subsequent pleural disease.

Here, we aim to use in vitro methods to elucidate the relative roles of the macrophages and mesothelial cells in driving the inflammatory response to long fibres. Long and short CNT samples were examined in an effort to infer whether the response to the CNT was length-dependent, as is the case in the inflammatory response in the pleural space in vivo [19]. Using the same panel of CNT as described by Murphy et al. [19] we examined the ability of long and short CNT to elicit pro-inflammatory responses in both a human non-transformed mesothelial cell line (Met5A), and macrophages derived from the human monocyte cell line (THP-1) as a surrogate for pleural macrophages, by direct exposure to CNT. The release of TNFα, IL-6, IL-8 and IL-1β; acute phase pro-inflammatory cytokines was measured as an indicator of the pro-inflammatory potential of the CNT panel. In addition we hypothesized that the inflammatory response could be driven by cross-talk between the macrophages and mesothelial cells and so we also examined the effect of conditioned media from CNT-treated macrophages on the mesothelial pro-inflammatory response.

## Results

### Effect of direct exposure to CNT panel on mesothelial cell and macrophage viability

**Figure 1:**
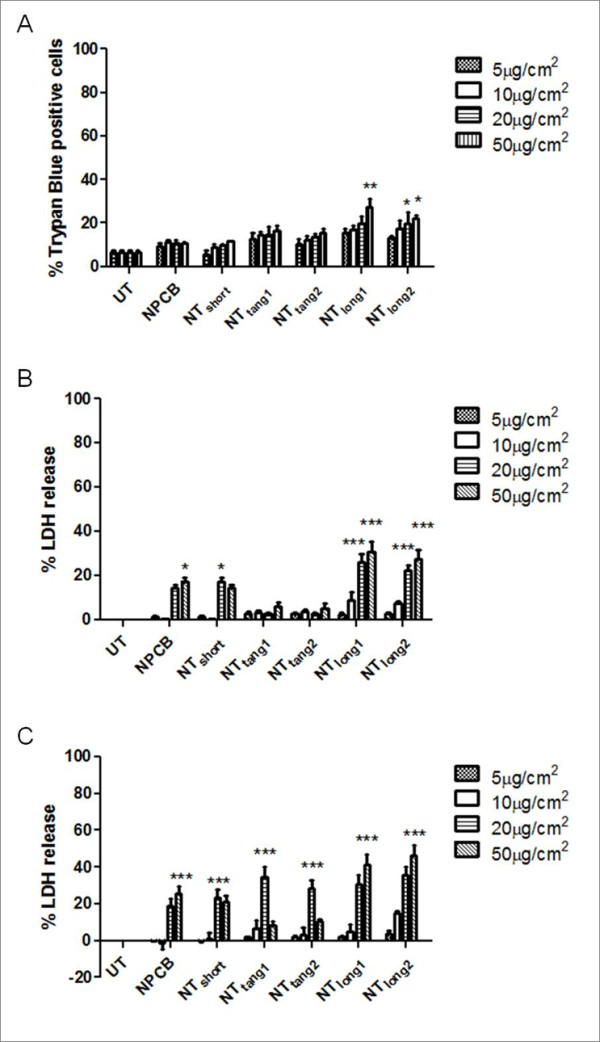
**The effect of the CNT panel on cell viability**. The viability of mesothelial cells was measured by trypan blue exclusion (A) and LDH release (B) after a 24 hour exposure to the CNT panel, UT indicates untreated control. LDH release was also used as an indicator of cell viability of macrophages at 24 hours after CNT treatment (C). Data expressed as a mean ± sem, n = 3. Significance versus vehicle control *** indicates *p *< 0.001.

Mesothelial cell viability, as measured by trypan blue exclusion assay, was examined after 24 hour exposure to the CNT panel over a dose range from 5 to 50 μg/cm^2^. A significant loss of cell viability was seen only in response to NT_long1 _(20 μg/cm^2^) and NT_long2 _(20 ug/cm^2^, 50 μg/cm^2^) (Figure 1A). This loss of cell viability coincided with an increase in supernatant LDH levels in Met5A cultures treated with NT_long1 _and NT_long2 _at the two highest doses only (Figure 1B). Exposure of macrophages (THP-1) to all the members of the CNT panel caused a significant increase in supernatant LDH only at the higher doses (Figure 1C). Trypan blue exclusion assay could not be carried out on THP-1 cells exposed to CNT as macrophages clumped together during the treatments making accurate assessment of the cellular viability by this method impossible. Based on these data a sub-lethal dose of 5 μg/cm^2 ^was selected for treatments of both Met5A and THP-1 cells for the subsequent activation studies.

### Effects of direct exposure to the CNT panel on pro-inflammatory cytokine release from mesothelial cells

**Figure 2:**
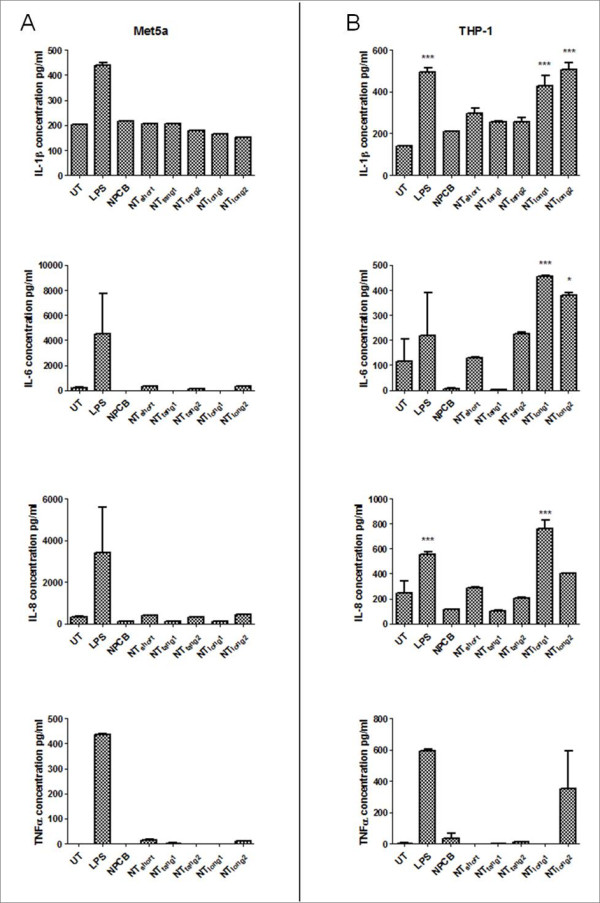
**The effect of the direct exposure to the CNT panel on cytokine release from mesothelial cells and macrophages**. No increase in the levels of IL-1β, IL-6, IL-8 or TNFα was detected after mesothelial cells were exposed to the CNT panel. However significant increases in IL-1β and IL-6 and IL-8 were seen in macrophages treated to long CNT samples only. UT indicates untreated control Data expressed as a mean ± sem, n = 3.

In order to investigate the effect CNT might have on mesothelial cell activation we assayed, using a cytometric bead array system, the concentration of a number of pro-inflammatory cytokines present in Met5A cell supernatants following direct exposure to the CNT panel for 24 hours (Figure 2A). No member of the CNT panel had an effect on the supernatant concentrations of IL-1β, IL-6, IL-8 or TNFα in Met5A cultures compared to LPS, a positive control to activate cells, which caused a significant increase in the concentration of all four cytokines.

### Effects of direct exposure to the CNT panel on pro-inflammatory cytokine release from macrophages

In order to investigate the impact of CNT phagocytosis on macrophage activation, we measured the concentration of IL-1β, IL-6, IL-8 and TNFα present in the THP-1 cells supernatants following exposure to the CNT panel (Figure 2B). Only the CNT samples containing long fibres- NT_long1 _and NT_long2 _caused a significant increase in IL-1β and IL-6 concentrations compared to untreated cells. NT_long1 _also induced a significant increase in IL-8 concentration; however no increase was seen with NT_long2_. NT_long2 _did appear to elevate the concentration of TNFα, but this elevation was not statistically significant. No increase in cytokine concentration was seen after exposure to NPCB, NT_short_, NT_tang1 _or NT_tang2_.

### Role of frustrated phagocytosis in length-dependent IL-1β release from macrophage

**Figure 3:**
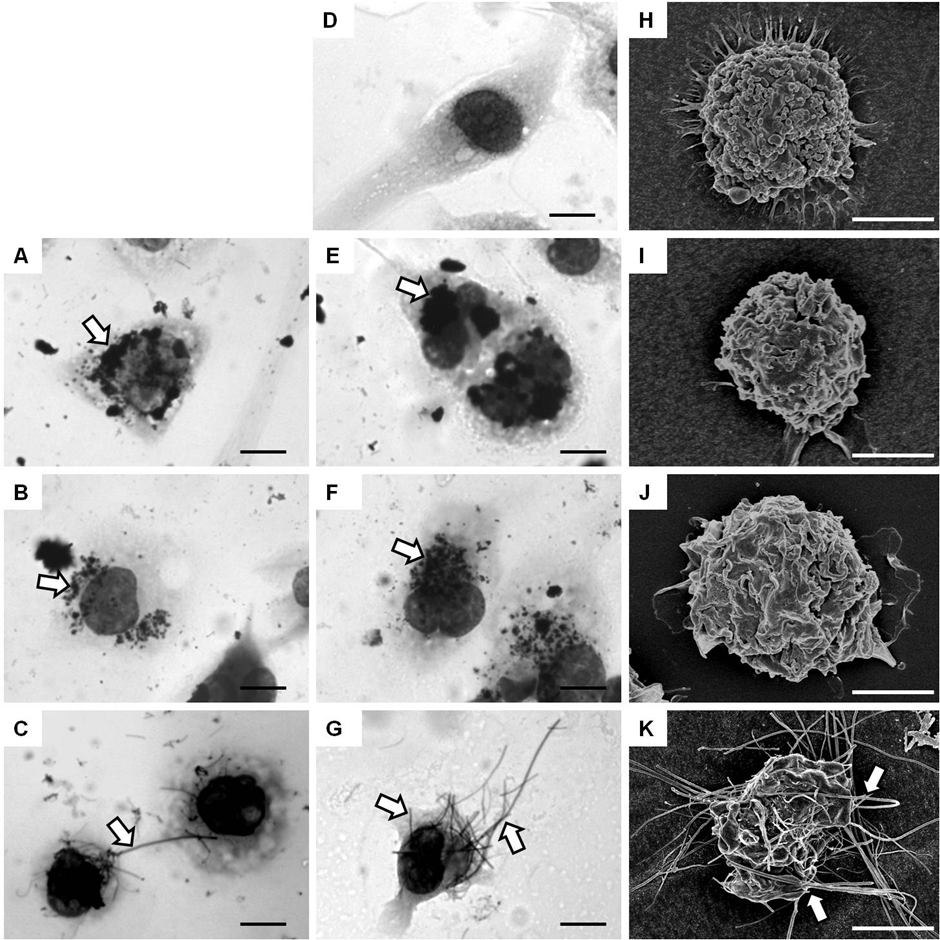
**Uptake of CNT by macrophages**. Light micrographs (A-G) of THP-1 cells untreated (D) or treated with the CNT panel. NPCB (A), NT_short _(E), NT_tang1 _(B) and NT_tang2 _(F) appear as aggregates within the cells (white arrows). However NT_long1 _(C) and NT_long2 _(G) appear to be protruding from the cells (white arrows). Scale bar indicates 20 μm. SEM images (H-K) show an untreated THP-1 cell (H) and THP-1 cells treated with NPCB (I), NT_short _(J) and NT_long2 _(K). No particles can be seen associated with the cell surface or protruding from the cells in I or J however the fibre from the NT_long2 _(K) sample appear on the surface of the cells and also protruding from the cell. Scale bar indicates 10 μm.

**Figure 4:**
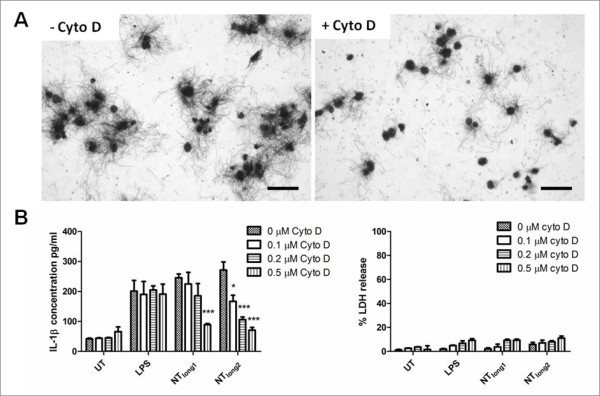
**Inhibition of IL-1β release from long CNT-treated macrophages**. (A) Example images of macrophages treated with NT_long2 _with or without cytochalasin D. Cytochalasin D inhibits the attempted uptake of long fibres by the macrophages. (B) Cytochalasin D caused a dose-dependent inhibition of IL-1β release from macrophages treated with NT_long1 _and NT_long2 _but not LPS. Significance versus 0 μM cytochalasin D within each treatment group *** indicates *p *< 0.001. A loss of cell viability in the presence of cytochalasin D was not detected by LDH release. Data expressed as mean ± sem, n = 3.

The appearance of the THP-1 cells after 24 hour treatment with the CNT panel at a dose of 5 μg/cm^2 ^is shown in Figure 3 as Diffquik stained light micrograph images (A-G) or as scanning electron micrograph figures (H - K). Normal cells are shown in Figure 3D and 3H. NPCB and the short CNT samples; NT_short_, NT_tang1 _and NT_tang2 _appear to be easily taken up by the macrophages as they are seen as accumulations of black particles within the cells (Figure 3A, B, E and 3F) but were not visible on the cell surface in SEM images confirming that they had been effectively phagocytosed (Figure 3I and 3J). NT_long1 _and NT_long2 _were not completely taken up by the cells which showed the classic features of frustrated phagocytosis with fibrous CNT extended from the cell surfaces (Figure 3G and 3K) or a single long fibre could be shared by more than one cell (Figure C). This state of frustrated phagocytosis appeared more extensive in cells treated with the longer NT_long2 _sample. We selected one cytokine, IL-1β, to test the hypothesis that the length-dependent pro-inflammatory effects were due to frustrated phagocytosis of long fibres. THP-1 cells were co-incubated with the NT_long1 _and NT_long2 _samples and an inhibitor of phagocytosis (cytochalasin D) and the release of IL-1β was measured by ELISA (Figure 4). Cytochalasin D, a potent inhibitor of actin polymerisation, prevented the uptake of the long CNT by the THP-1 cells (Figure 4A) and inhibited the increase in IL-1β concentration caused by exposure to NT_long1 _and NT_long2 _in a dose-dependent pattern. However it had no effect on the IL-1β concentrations caused by LPS, which does not require phagocytosis to elicit a pro-inflammatory response. Measurements of supernatant LDH levels showed there was no loss of cell viability due to cytochalasin D treatments (Figure 4B).

### Effect of conditioned media from CNT-exposed macrophages on mesothelial cell viability

**Figure 5:**
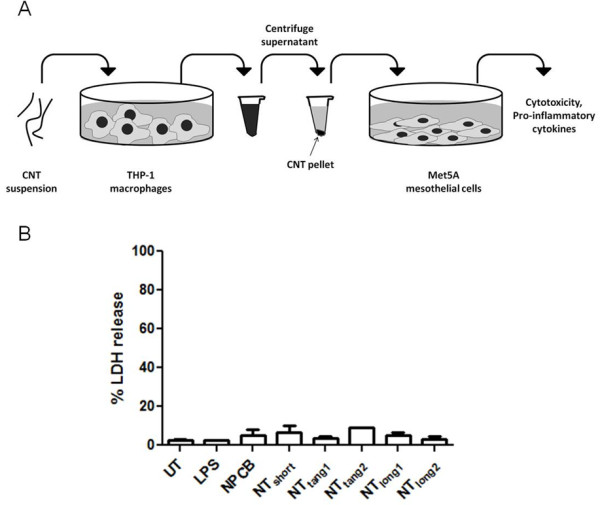
**The effect of conditioned media from CNT-exposed macrophages on mesothelial cell viability**. (A) Schematic diagram representing the conditioned media experimental procedure. THP-1 cells were treated with CNT suspensions for 24 hours, supernatant was removed and centrifuged to remove any suspended CNT, conditioned media was added to the Met5A cells for 24 hours after which cell viability and production of cytokines was measured. Cell viability of mesothelial cells was measured by LDH release (B) but no cell death was detected.

In order to test the hypothesis that CNT that reach the pleural space stimulate mesothelial cells indirectly via the release of cytokines from particle-activated macrophages, we used conditioned media from CNT-exposed THP-1 cells to treat Met5A cells and measured the concentrations of pro-inflammatory cytokines in the Met5A supernatant (Figure 5A).

No increase in LDH release from the Met5A cells treated with the conditioned media confirm the treatments did not caused a loss of membrane integrity and therefore cell viability (Figure 5B).

### Effect of conditioned media from CNT-exposed macrophages on mesothelial cell production of pro-inflammatory cytokines

**Figure 6:**
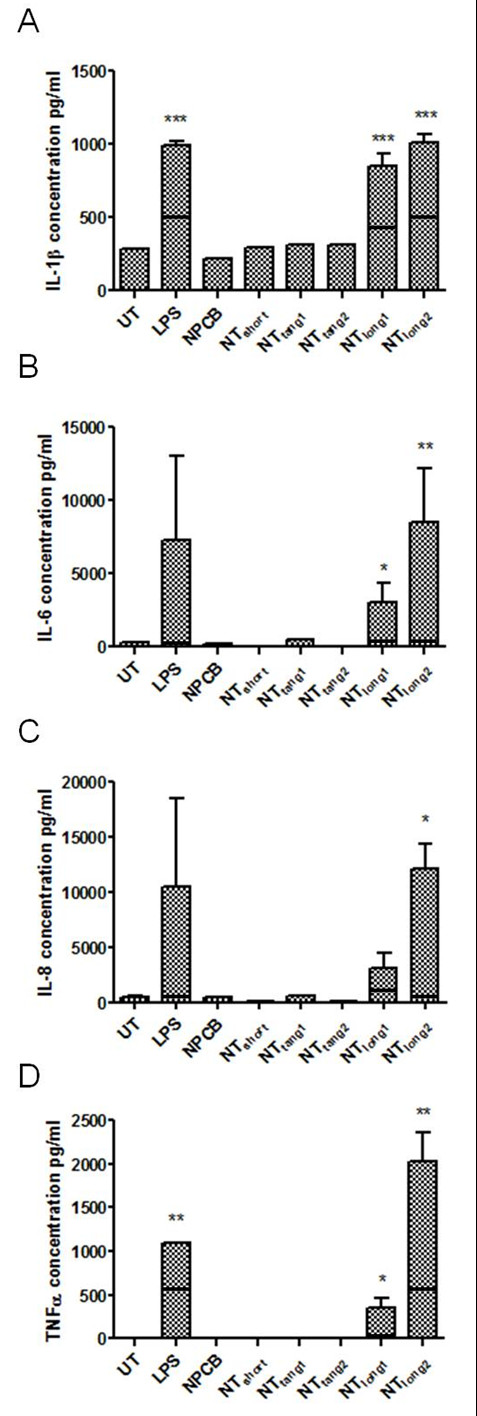
**The effect of conditioned media on cytokine release from cells**. IL-1β, IL-6, IL-8 and TNFα supernatant levels were measured 24 hours after mesothelial cells were exposed to media from CNT-treated macrophages. A length-dependent increase in concentration was observed for each of the cytokines. The black horizontal lines on the graph indicate the level of cytokine expressed by the THP-1 cells which is still present in the conditioned media. Data expressed as mean ± sem, n = 3. Significance versus vehicle control *** indicates *p *< 0.001.

Whilst direct exposure of Met5A cells to the CNT panel had not caused any increases in IL-1β, treatment with conditioned media from THP-1 cells exposed to LPS, NT_long1 _or NT_long2 _resulted in approximately two-fold increase in IL-1β concentration compared with the combined total from both cell types exposed directly as shown by the horizontal lines on the bars (Figure 6A).

Media from THP-1 cells exposed to the NT_long1 _and NT_long2 _samples also had a significant effect on the production of IL-6, IL-8 and TNFα by Met5A (Figure 6B-D). The potentiation of Met5A activation was most pronounced when Met5A had been exposed to NT_long2_-exposed THP-1 media which resulted in a 20-fold, 30-fold and 6-fold increase in the concentrations of IL-6, IL-8 and TNFα respectively, when compared with the combined total from each cell type exposed directly. Conditioned media from NT_long1 _treated THP-1 cells also caused a significant increase in the production of IL-6, IL-8 and TNFα from Met5A cells albeit to a lesser extent. Exposure to media from THP-1 cells treated with NPCB, NT_short_, NT_tang1 _or NT_tang2 _did not have any effect on the production of the pro-inflammatory cytokines; IL-1β, IL-6, IL-8 or TNFα, by Met5A cells.

**Figure 7:**
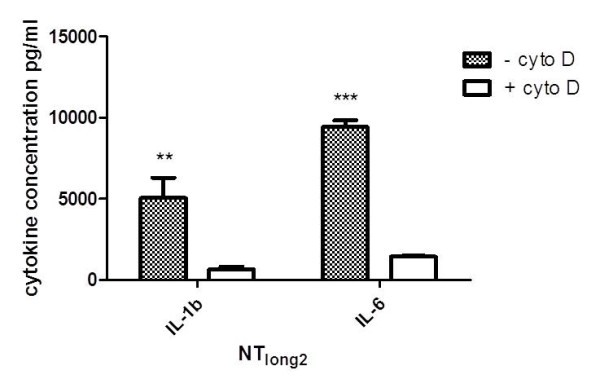
**The effect of conditioned media from macrophages co-exposed to NT_long2 _and cytochalasin D on mesothelial production of IL-1β and IL-6**. A significant reduction in the levels of IL-1β and IL-6 release from mesothelial cells exposed to conditioned media from macrophages treated with NT_long2 _and cytochalasin D was observed compared to mesothelial cells treated with conditioned media from macrophages treated with NT_long2 _alone. Data expressed as mean ± sem, n = 3. Significance versus vehicle control *** indicates *p *< 0.001.

The levels of IL-1β and IL-6 released from Met5A cells treated with the conditioned media from THP-1 cells treated with NT_long2 _was attenuated when the THP-1 cells were co-exposed to NT_long2 _and cytochalasin D to prevent phagocytosis (Figure 7).

#### Iron content

The iron content of the CNT samples is given in the Additional file 1.

#### Interference of CNT in the assays

We precluded interference of CNT in the assays used here and the evidence is present in the Additional file 1.

## Discussion

**Figure 8:**
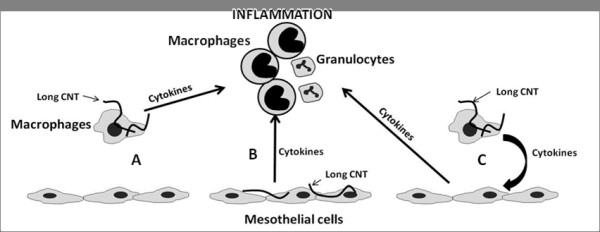
**Potential mechanisms of the long fibre-mediated inflammatory response**. The following three potential mechanisms by which CNT cause inflammation in the pleural space were explored in this study. (A) Long fibres interact with the pleural macrophages producing cytokines that directly elicit an inflammatory response. (B) Long fibres interact directly with mesothelial cells with the subsequent factors released leading to granulocyte influx. (C) Long fibres interact with macrophages inducing the release of cytokines which stimulate the mesothelial cells, amplifying the inflammatory response. As initial results showed no response from the mesothelial cells to direct exposure to CNT, the effect of factors released from CNT-exposed mesothelial cells on macrophages was not examined.

We previously reported that long CNT but not short CNT were capable of causing inflammation in the peritoneal [17] and pleural spaces [19]. Whilst retention of the long fibres at stomata represent the key initiating step the cellular mechanism, especially the interaction between macrophages and mesothelial cells, leading to inflammation was not elucidated. The present study addressed three hypotheses as to the mechanism by which CNT cause pro-inflammatory effects in the pleural cavity. These three hypotheses centred on the major cells of the pleural cavity, mesothelial cells and macrophages, are outlined in diagrammatic form in Figure 8. The refractoriness of the mesothelial cells to long CNT effects and the existing literature describing how conditioned media from macrophages treated with particles greatly amplify the pro-inflammatory responses of epithelial cells [22] and endothelial cells [23] led us to discount the alternative hypothesis, that products released by mesothelial cells could stimulate amplifying pro-inflammatory responses in the macrophages. Whilst mesothelial cells did prove refractory, in terms of cytokine release, to direct exposure to long and short CNT, macrophages exposed to long fibre CNT samples did release significant quantities of pro-inflammatory cytokines. When the supernatant from the long fibre-exposed macrophages was added to mesothelial cells it stimulated the mesothelial cells to release substantial quantities of cytokines that were much greater than those produced by the macrophages in response to long CNT or the mesothelial cells exposed to long CNT (Summarised in Table 1) or the sum of these two. Therefore the pro-inflammatory milieu generated by macrophages in response to long fibre CNT samples was a potent activator of mesothelial cells and caused a dramatic amplification of cytokine production by the mesothelial cells. Supernatants were centrifuged at 13,000 rpm for 5 minutes to remove any CNT and this procedure produced a black pellet and a clear supernatant. When the clear supernatant was added to the target mesothelial cells, in contrast to the direct treatment with CNT where black CNT were readily visible inside the cells, no particles were visible in or on the cells. It is not possible to preclude the presence of very small amounts of CNT but these would be trace amounts well below the threshold of effect.

**Table 1:** Summary of cytokine release from cells

	IL-1β		IL-6		IL-8		TNFα	
	**NT_short_**	**NT_long2_**	**NT_short_**	**NT_long2_**	**NT_short_**	**NT_long2_**	**NT_short_**	**NT_long2_**

Mesothelial cells (pg/m)	205 **± **26	153 **± **17	340 **± **11	347 + 2	392 **± **15	458 **± **16	15 **± **2	10 **± **3

Macrophages (pg/ml)	296 **± **23	506 **± **34	128 **± **39	380 + 8	286 **± **8	403 **± **4	6 **± **2	350 **± **200

Mesothelial cells + conditioned media (pg/ml)	293 **± **2	1012 **± **59	214 **± **8	8479 + 3640	121 **± **29	12130 **± **2254	14 **± **4	2029 **± **328

The cytokine release for the three treatment groups are compared side by side using one short CNT (NT_short_) and one long CNT (NT_long2_) as exemplars

The mechanism and outcome of phagocytosis of long CNT differs from that of short CNT or NPCB, in that phagocytosis proceeds initially but the macrophage cannot effectively enclose the extended structure of a long fibre culminating in the phenomenon of 'frustrated phagocytosis' [24]. The uptake of long fibres by macrophages and the subsequent pro-inflammatory response from the mesothelial cells exposed to their supernatant could be attenuated by inhibiting the phagocytosis of the long fibres by macrophages during the initial treatments using the microfilament poison Cytochalasin D; this confirms that the process of frustrated phagocytosis is is the key one that leads to inflammation in the case of the long fibre CNT. The macrophage response to LPS, which does not require microfilament-mediated uptake into cells by phagocytosis, was not attenuated by the addition of cytochalasin D highlighting the necessity of particle uptake and the role of frustrated phagocytosis in the pro-inflammatory reaction produced in response to CNT exposure. Our results suggest that indirect activation of mesothelial cells by pro-inflammatory cytokines elaborated from longCNT-exposed macrophages is a key initiating event in the development of pleural inflammation and disease.

Previous studies have described that long, needle-like CNT and asbestos activated the NLRP3 inflammasome and secretion of IL-1β from LPS-primed human macrophages [25] whilst our macrophages were not primed with LPS. In our study the THP-1 cells had been differentiated with PMA [26]which gives then a phenotype similar to resting monocyte-derived macrophages.

A proportion of all particles and fibres that deposit in the distal regions of the lung will translocate into the pleural cavity thereby facilitating direct interactions between them and the patrolling macrophages of the pleural space and the mesothelial cells lining it [24]. According to Mercer et al. [34]CNT that reach the distal lung are likely to be predominantly present inside alveolar macrophages. However CNT in the immediate sub-pleural site, whilst a low proportion of all CNT, were all in tissue i.e. not in macrophages [27]. Therefore the CNT in the pleural space may be taken there inside alveolar macrophages, whilst the non-phagocytosed CNT that reach the pleural space from the sub-pleural tissue may be taken up by pleural macrophages. Research is needed to better understand the secretory profile of pleural macrophages or alveolar macrophages containing CNT in the pleural space. Mesothelial cells are reported to be uniquely sensitive to asbestos fibres with direct exposure in vitro inducing death [28,29], inflammatory mediator production [28], oxidative stress [30] and genotoxicity [39,40]. Although the potential pathogenicity of CNT has been tested in a variety of lung-derived cell types with responses including inflammation [33] and oxidative stress [34] reported, there is a paucity of data concerning the direct effects of CNT on mesothelial cells. Pacurari et al. examined the direct effects of SWCNT and MWCNT on normal and malignant mesothelial cells and reported a decrease in cell viability, activation of pro-inflammatory transcription factors and pro-inflammatory MAPK pathway activation [35,36]. However these effects were very modest and seen at 5-10 times the dose used in the present paper. There was also ongoing PARP activation suggesting that the cells were also undergoing apoptosis, making this data difficult to interpret as to the true role of direct pro-inflammatory effects of CNT on mesothelial cells at plausible doses. In contrast, within the present study we found mesothelial cell cytotoxicity only at the highest exposures used in preliminary studies but no evidence of pro-inflammatory responses after exposure of mesothelial cells to a low sub-lethal dose (5 μg/cm^2^) of the panel of CNT. The inflammatory response to CNT after direct injection into the pleural space in vivo, characterised by an influx of inflammatory granulocytes [19], would undoubtedly require the actions of acute phase cytokines such as IL-1β, TNFα, IL-6 and IL-8. The inability of CNT to directly stimulate the production of such cytokines in mesothelial cells in vitro would suggest the interactions between CNT and mesothelial cells are not directly responsible for the initiation of inflammation.

In contrast to the findings with mesothelial cells, exposure of macrophages to the CNT panel resulted in the induction of modest length-dependent pro-inflammatory responses. This was evident when only the long fibre-containing samples (NT_long1 _and NT_long2_) induced the production of the acute phase cytokines IL-1β and IL-6. The lack of response to the short CNT samples and particulate graphite in the form of NPCB suggest that the pro-inflammatory response to CNT in the pleural space is solely dependent on length. Length is a controlling factor in the clearance of fibres from the pleura where only long fibres are selectively retained for sufficient time to actually become involved in interactions with macrophages at stomatal openings [19]. Any fibre that exceeds a maximum length for macrophage uptake will result in frustrated phagocytosis, a state where the macrophages are unable to fully engulf long fibres resulting in the release of oxidants and pro-inflammatory signals [24]. Frustrated phagocytosis has long been known to play an important role in asbestos effects [24,37] and has also been implicated more recently in the inflammatory responses to long CNT elicited from macrophages in vitro [38] and in vivo [17] and long TiO_2 _nanofibres [39]. Brown et al. reported an increase in the production of TNFα and a concurrent increase of ROS by macrophages in response to CNT and showed that this response was specifically related to the length of the CNT fibres [38]. The presence of macrophages undergoing frustrated phagocytosis in the peritoneal lavage fluid of mice exposed to long CNT was noted by Poland et al. who suggested that this macrophage-mediated response may be playing a role in the inflammation [17]. Here, using a panel of CNT similarly defined by length we showed the length-dependent release of a number of pro-inflammatory cytokines from macrophages exposed to the long fibre CNT samples only. Frustrated phagocytosis of TiO_2 _nanobelts longer than 15 μm was demonstrated by Hamilton et al. who also described the mechanism by which the inability of macrophages to completely enclose long fibres lead to pro-inflammatory responses via lysosomal destabilisation and NALP3 inflammasome activation [39]. The NALP3 inflammasome controls the maturation and release of IL-1β from activated cells [40] and activation of the NALP3 inflammasome via lysosomal destabilisation and the release of the lysosomal enzyme cathepsin B was reported in response to macrophage uptake of crystalline silica particles [41]. The activation of the NALP3 inflammasome by long CNT has also recently been demonstrated by Palomaki et al. in primary macrophages primed with LPS who, also reported a role for the P2X_7 _receptor and its downstream tyrosine kinases, Src and Syk, in the activation of the NALP3 inflammasome in response to 'rigid, needle-like material' [42]. The induction of NALP3 via frustrated phagocytosis of long CNT is in keeping with studies examining the inflammatory response to asbestos and other fibrous particles which have also demonstrated the importance of the NALP3-mediated inflammatory response to particles and fibres in vivo. Inhibition or knock-out studies of key components of the NALP3 inflammasome prior to administration of asbestos fibres [43], silica [41,43,44] or MSU [41] crystals into the lungs of mice have all shown an attenuated inflammatory response compared to wild-type controls. The role for NALP3 in the activation of an inflammatory response suggests that direct interaction between macrophages and long fibres is the initiating mechanism for the development of long fibre-related inflammation and subsequent disease. Mesothelial cells are well equipped to participate in the initiation and resolution of inflammation. Secretion of chemokines by stimulated mesothelial cells promotes directed migration of granulocytes which can lead to influx of inflammatory cells from the vasculature into the serosal space [45]. Mesothelial cells have been shown to be directly stimulated to produce a range of pro-inflammatory mediators including cytokines, chemokines, growth factors and oxidants in response to bacterial endotoxin and asbestos but have also been shown to be highly responsive to factors secreted by macrophages [20]. Previous studies with PM10 and diesel soot have also shown that fixed cells- epithelial cells and endothelial cells respectively, show much greater responses to particle-free conditioned media from macrophages treated with particles than to direct treatment with the particles [22,23]. Similarly, in the present study a greatly amplified mesothelial pro-inflammatory response was conferred by the particle-free conditioned medium of long CNT-exposed macrophages. The role of cross-talk between macrophages and mesothelial cells in amplifying inflammation has been demonstrated previously by Betjes et al. when peritoneal mesothelial cells were shown to produce high levels of IL-8 in response to conditioned media from macrophages treated with the bacteria *Staphylococcus epidermidis*, but not to the bacteria themselves [46]. IL-1β and TNFα also mimicked induction of the mesothelial IL-8 response and the response to the conditioned media was blocked by the addition of anti-IL-1β and anti-TNFα antibodies [46]. The pro-inflammatory effect of the supernatant from macrophages exposed to the long CNT samples described here was similarly attenuated by inhibiting release of IL-1β by blocking phagocytosis with cytochalasin D highlighting the importance of phagocytosis in eliciting secretion of cytokines that drive the pro-inflammatory effects on the mesothelial cells. The acute inflammatory response seen in the pleural space after the injection of long CNT is characterised by the rapid influx of granulocytes into the pleural cavity [19]. The data suggest that it is a release of high levels of mesothelial IL-8 or KC, the mouse analogue of IL-8 in response to stimulatory factors released from macrophages attempting to phagocytose long CNT fibres that explains the inflammatory cell influx in vivo. Taking these 3 studies together [22,23,46] TNFα and IL-1β are the most likely candidate cytokines released by macrophages undergoing frustrated phagocytosis of long CNT that drive the pro-inflammatory effects in the mesothelial cells seen in our studies.

The mesothelial response to macrophage stimulation is also important in tissue repair. Macrophages were recruited in large numbers to the pleural space following long CNT deposition there [19]. Macrophages have been shown to stimulate mesothelial cell proliferation in response to injury in vivo with the rate of serosal healing dependent on the number of macrophages present [47] and also the macrophage-mediated release of cytokines such as TNFα [48]. Our data suggest that macrophages exposed to long CNT in the pleural space will have similar mitogenic effects on mesothelial cells which may lead to disregulated growth patterns.

The cross-talk between macrophages and mesothelial cells is known to be important in the normal inflammatory processes in the serosal cavities but, if disregulated, may also help to promote the development of mesothelioma. The precise mechanism that leads to mesothelioma development in the presence of fibres is unknown but a role for chronic inflammation in response to retained biopersistent fibres has been postulated [15]. Exposure of normal human mesothelial cells to asbestos fibres in vitro has not been shown to lead to cell transformation even though phenotypic changes such as chromosomal changes and extended lifespan were observed [31,32]. However Wang et al. [49] carried out a study examining the effect on mesothelial cells of erionite, a naturally occurring long biopersistent fibre which causes mesothelioma in man and rodents following inhalation [50-52]. In this study, treatment of mesothelial cells with erionite in combination with pro-inflammatory cytokines highlighted a potential role for an inflammatory environment in the transformation of mesothelial cells [49]. While erionite alone had no effect on cell transformation, both IL-1β and TNFα could stimulate formation of transformed cells as identified by their anchorage-independent growth in soft agar. While the cytokines could induce the formation of tumorigenic colonies alone the effect was more potent in the presence of the erionite fibres [49]. TNFα has also been shown by Yang et al. to inhibit asbestos-induced cell death by activating the NF-κB signalling pathway in mesothelial cells [53]. The TNFα-mediated resistance to the cytotoxicity may promote tumour formation by increasing the pool of mesothelial cells with fibre-mediated genomic damage in the inflammogenic environment which evade normal cell death. This suggests that inflammation and inflammatory cytokines may play an important role in mesothelioma. Here we showed that mesothelial cells exposed to a cocktail of cytokines and other mediators produced by macrophages treated with long CNT amplify and propagate the inflammatory response thereby likely contributing to disease development in the pleural space.

A recent paper [54] has focused on the diameter of CNT and claimed that MWCNTs ~50 nm diameter showed mesothelial cell membrane piercing and cytotoxicity in vitro and subsequent inflammogenicity; in contrast CNT with a diameter of ~150 nm or ~2-20 nm were less inflammogenic. We found no support for this diameter hypothesis - the samples with least activity (short or tangled CNT) had diameters of 14.8, 10.4 and 84.9 nm respectively whilst the most active (the long CNT) was 165 nm diameter, quite close to the 150 nm diameter found to have no activity in the Nagai study [54].

Iron has been implicated in CNT activity via it's ability to cause oxidative stress [55,56]. However the levels of iron in our sample showed no relationship with activity in any of the assays; in particular the long CNT which had the most activity, had least iron.

Although we deal here solely with inflammation as a factor in fibre pathogenicity and mesothelioma production there are other effects of long fibres on mesothelial cells such as clastogenic and genotoxic ones that are important in themselves and possibly in concert with inflammation in leading to mesothelioma [54,57].

## Conclusion

In summary, the data presented in this study describe a mechanism for the initiation of a long fibre mediated inflammatory response in the pleural space via frustrated phagocytosis. Only long fibres are retained in the pleural space and we have shown firstly that the incomplete phagocytosis of long CNT by macrophages, but not mesothelial cells, elicits a modest pro-inflammatory cytokine response. Secondly we show that the supernatant from macrophages exposed to long fibres produces a much-amplified pro-inflammatory response in target mesothelial cells. This amplified mesothelial cell response to macrophage products was attenuated by inhibition of macrophage phagocytosis, of long fibres confirming a role for frustrated phagocytosis. This study furthers our understanding of the role of macrophage/mesothelial cross-talk as a mechanism underlying the generation of a length-dependent inflammatory response to CNT in the pleural space. In addition, this in vitro model may prove useful for in vitro toxicity screening of the large number of new high aspect ratio nanofibres currently being developed, thereby reducing the need for animal testing.

## Methods

### CNT panel

The panel of particles investigated consisted of 5 different samples of multiwalled CNT and nano-particle carbon black (NPCB) as was used previously in Murphy et al. [19] (Table 2). The NT_long1 _sample (Mitsui & Co. Ltd., Japan) was produced by catalytic chemical vapour synthesis using the floating reaction method. The NT_long2 _sample was produced in an academic research laboratory (Dr Ian Kinloch, University of Manchester) using catalytic vapour discharge (CVD) method using a ferrocene-toluene feedstock to grow nanotubes from iron catalysts held on a silica plate. These nanotubes grew aligned as mats, meaning they were straight and un-entangled. The nanotubes were harvested from the mats using a razor blade, with some residual iron remaining within the nanotubes. We also included one commercially available short straight CNT (NT_short; _Nanoamor Inc., TX, USA) and two curled and tangled nanotubes of different lengths (NT_tang1 _which was cut to form predominantly short NT fibres and the original length NT sample (NT_tang2_); NanoLab, Inc., MA, USA). These were produced by CVD with an iron and ceramic oxide (alumino-silicate) catalyst support which was removed using HCl and Hydrofluoric acid treatment. Trace metals and endotoxin levels previously tested and reported in Poland et al. [17] were low and thus not considered to play a role in these studies.

**Table 2:** Characteristics of the particle panel

	NPCB	NT_short_	NT_tang1_	NT_tang2_	NT_long1_	NT_long2_
**Source**	**Degussa Printex 90**	**Nanostructured & Amorphous Materials, Inc**.	**NanoLab, Inc**.	**NanoLab, Inc**.	**Mitsui & Co**.	**University of Manchester** **[Dr. I. Kinloch]**

Diameter (nm)	14	25.7 ± 1.6	14.84 ± 0.05	10.40 ± 0.32	84.89 ± 1.9	165.02 ± 4.68

Length (μm)	-	1-2	1-5	5-20	Mean 13	Mean 36

% fibre greater than 15 μm	‡	‡	‡	‡	24.04	84.26

### CNT suspensions

CNT were suspended in RPMI-1640 media (PAA Laboratories Ltd., UK) containing 0.5% bovine serum albumin (BSA; Sigma-Aldrich, Poole, UK) at a concentration of 500 μg/ml and dispersed by sonication at 230 V, 50 Hz, 350 W for 2 hours in an ultrasonic bath (FB11002, Fisherbrand, Thermo Fisher Scientific, Inc., MA, USA). Suspensions were prepared freshly each day and used immediately upon removal from the ultrasonic bath.

### Cell culture and treatment

The immortalised human mesothelial cell lineMet5A, and the monocytic cell line THP-1 were obtained from the American Type Culture Collection (ATCC) and maintained at sub-culture in RPMI-1640 supplemented with 10% foetal calf serum (PAA Laboratories Ltd., UK) at 37°C and 4% CO_2_. Prior to experimentation Met5A cells were seeded in 24-well plates (Corning, Amsterdam, The Netherlands) at a concentration of 2.5 × 10^5 ^cells/well and allowed to adhere for 24 hours. THP-1 monocytic cells (2.5 × 10^5 ^cells/well) were differentiated into macrophages with 10 ng/ml phorbol 12-myristate 13-acetate (PMA; Sigma-Aldrich, Poole, UK) in 24-well plates for 48 hours. Prior to the treatment of both cell types the media was replaced with RPMI media containing 0% FCS, 1% penicillin/streptomycin and 1% L-Glutamate. Cells were treated with the CNT panel for 24 hours using a range of doses to determine cell viability, 5 μg/cm^2 ^was chosen as a sub-lethal dose for subsequent activation studies. Cytochalasin D (Enzo Life Science) was used to co-treat THP-1 cells along with NT_long1_, NT_long2_, or LPS. Light microscopy images of THP-1 cells treated with the panel of CNT were captured at ×40 magnification using QCapture Pro software (Media Cybernetics, MD, USA). For the conditioned media treatments THP-1 cells were treated with the CNT panel (5 μg/cm2) for 24 hours, supernatant was removed and centrifuged at 13,000 rpm for 5 minutes to remove any CNT. Conditioned media was added to the Met5A cells for 24 hours.

### Scanning electron microscopy

THP-1 cells were grown on Thermonox coverslips (Nunc, Roskilde, Denmark) and treated with NPCB, NT_short _or NT_long2 _for 24 hours. Cells were fixed with 10% Formalin and were stained with osmium tetroxide prior to critical point drying, mounted and gold sputter coated before examination by scanning electron microscopy (SEM) using an Hitachi S-2600 N digital scanning electron microscope (Oxford Instruments, Oxfordshire, UK).

### Trypan blue exclusion assay

Met5A cells were plated as above before treatment with the particle panel for 24 hours at doses ranging from 5-50 μg/cm^2^. The cell supernatant was removed and kept for LDH measurements, cells were washed once with PBS and incubated with 0.4% trypan blue (Sigma-Aldrich, Poole, UK) for 5 minutes. Excess trypan blue was removed and cells washed with PBS. Dead cells, as indicated by incorporation of the trypan blue dye, were counted and calculated as a percentage of total cells.

### Lactate dehydrogenase assay

One hundred microlitres of cell supernatant from Met5A and THP-1 cells exposed to the CNT panel at doses ranging from 5-50 μg/cm^2 ^or LPS (1 μg/ml) was added in triplicate to a 96 well plate (Corning, Amsterdam, The Netherlands) and 100 μl of the LDH test reagent (diaphorase/NAD + mixed with iodotetrazolium chloride and sodium lactate at a ratio of 1:45) added to each well. Cells treated with 0.1% Triton-X were used as a positive control for 100% cell lysis. Following a 30 minute incubation period the absorbance of each well at 490 nm wavelength was established using a Synergy HT microplate reader (BioTek Instruments, Inc. VT, USA).

### Cytokine bead array

The media levels of IL-1β, IL-6, IL-8 and TNFα were measured after direct exposure of the mesothelial cells and macrophages to the CNT panel and exposure of the mesothelial cells to the conditioned media from CNT-treated macrophages by cytokine bead array (BD CBA Flex Set, BD Biosciences, San Jose, CA). Briefly, 25 μl of the mixed capture antibodies were added along with 50 μl of the supernatant samples and standards to each well of a 96-well plate and incubated at room temperature for one hour. Twenty-five microlitres of the mixed PE detection reagent was added to each well and incubated at room temperature for two hours. The plate was centrifuged at 1500 rpm for 5 minutes and the supernatant completely removed. One hundred and fifty microlitres of the wash buffer was added to each well. The plate was agitated for 5 minutes to resuspend the beads before the samples were analyzed using the BD FACSArray Bioanalyzer (BD Biosciences, San Jose, CA). Results were analysed using FCAP array software and sample concentrations of each cytokine were established via extrapolation from the appropriate recombinant protein standard curve.

### ELISA

The media levels of IL-1β and IL-6 after macrophage inhibition studies was established using ELISA DuoSet kits (R&D systems, Abingdon, UK) specific to each analyte of interest. Ninety-six well microtitre plates were incubated overnight at 4°C with 100 μl of coating antibody raised against IL-1β or IL-6. The plates were washed 3 times with 0.05% Tween-20 in phosphate buffered saline (PBS; pH 7.2) and blocked using reagent diluent (1% BSA in PBS; R&D systems, Abingdon, UK) for 1 hour (room temperature) prior to further washing and addition of test samples/standards in triplicate. After 2 hrs the plates were washed and a biotinylated detection antibody added to each well followed by a further 2 hr incubation, followed by washing and the addition of HRP conjugated Streptavidin. The plates were washed and developed using a TMB substrate solution (Sigma-Aldrich, Poole, UK). The subsequent reaction was stopped with 0.5 M H_2_SO_4_, resulting in a yellow colour, and read at 450 nm. Sample concentrations of IL-1β, IL-6 were established via extrapolation from the appropriate recombinant protein standard curve.

### Statistical analysis

All data are shown as the mean + s.e.m. and these were analysed using one-way analysis of variance (ANOVA). Multiple comparisons were analysed using the Tukey-HSD method, with values of *P *< 0.05 considered statistically significant (Instat, Graphpad Software Inc., CA, USA).

## Abbreviations

CNT: carbon nanotubes; THP-1: macrophage cell line; Met5A: mesothelial cell line; NPCB: nanoparticulate carbon black; PARP: Poly (ADP-ribose) polymerase; TiO2: titanium dioxide; NALP3: NACHT: LRR and PYD domains-containing protein 3; P2X7: purinergic receptor; MSU: monosoium urate; IL-8: Interleukin 8; KC: keratinocyte -derived chemokine; TNFα: tumour necrosis factor α; NF-κB: Nuclear factor-κB; CVD: catalytic vapour discharge; ATCC: American Type Culture Collection; RPMI: Roswell Park Memorial Institute Medium; FCS: foetal calf serum; SEM: scanning electron microscopy; PBS: phosphate buffered saline; LDH: lactate dehydrogenase; FACS: fluorescence activated cell sorter.

## Competing interests

KD has carried out consultancy for the nanotechnology industry not related to the scientific content of this manuscript.

## Authors' contributions

All authors have made substantial contributions to conception and design and interpretation of data; FAM acquired the data with the assistance of AS. All authors were involved drafting or revising the manuscript and all authors have given final approval of the version to be published. All authors take public responsibility for the content. All authors read and approved the final manuscript.

## Supplementary Material

Additional file 1 **Supplementary information**. Click here for file
