# Supplementary material for: The mechanism of pleural inflammation by long carbon nanotubes: interaction of long fibres with macrophages stimulates them to amplify pro-inflammatory responses in mesothelial cells
Source: Part Fibre Toxicol. 2012 Apr 3;9:8. doi: 10.1186/1743-8977-9-8 (PMC3352110; doi:10.1186/1743-8977-9-8)
Supplement: Additional file 1 — Supplementary information. [file 1743-8977-9-8-S1.doc]

Murphy et al

Supplementary material

Excluding CNT interference in the assay

We checked the potential of the CNT samples used here to interfere with assays where elements of the assay might adsorb to the particle surface. To test for an effect in the LDH assay Met5A cells were lysed (100% LDH release) and incubated with 50μg/cm^2^ long/short CNT or media control and LDH assessed.. No difference was seen between the LDH levels in samples incubated with the media control or the CNT samples (Supplementary Figure 1). The Trypan blue assay is not amenable to adsorption artefact since the blueness of cell is estimated by eye and, cells were not sufficiently highly loaded that the blue staining was obscured. In addition, the similarity in results between the Trypan Blue assay and the LDH assay with only the long CNT proving to have statistically significant cytotoxicity supports the contention that the cytotoxicity assays are not affected by adsorption. In order for adsorption artefact to explain the difference between NT long and NTshort in cytokine assays the NTshort would need to adsorb 3 - 6 times more cytokine per unit mass than the NTlong. We chose IL-8 as an examplar cytokine and diluted it to 500 pg/ml and dispersed NT long and NT short at 5μg/ml, the only dose used in these studies; these were then incubated overnight and IL-8 measured. There was no significant difference in the levels of IL-8 between untreated IL-8 and IL-8 treated with either long or short CNT (Supplementary Figure 2).

We conclude that interference of CNT in assays cannot explain any of the differential effects seen between long and short CNT in this study.

Iron content

The soluble iron content of the samples here has been previously published [1] and were as follows :- (units μg/g) CNT_short_ 24.3 , CNT_tang_ 7.9, CNT_long_ <0.1.


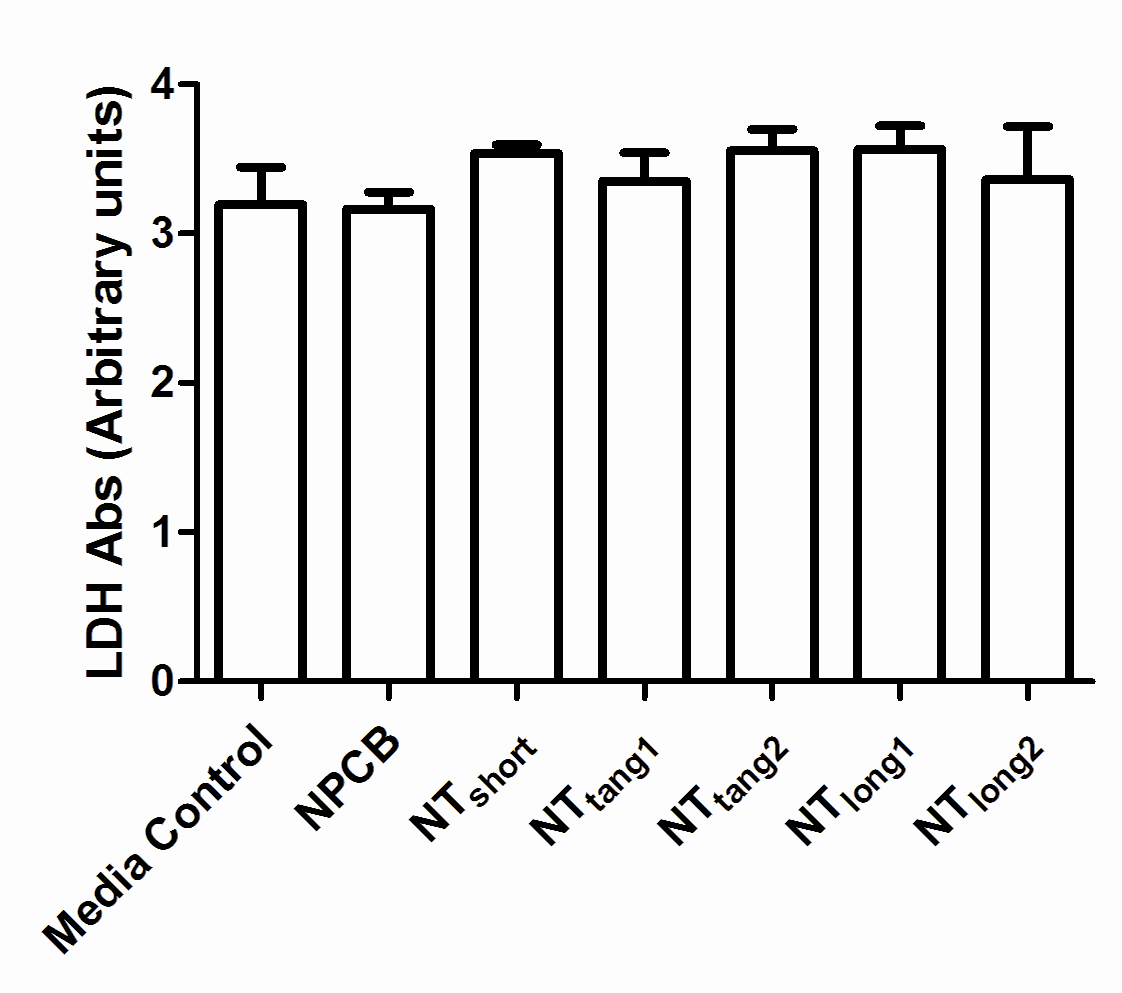


Supplementary Figure 1 Effect of CNT on measurement of LDH in the LDH assay.


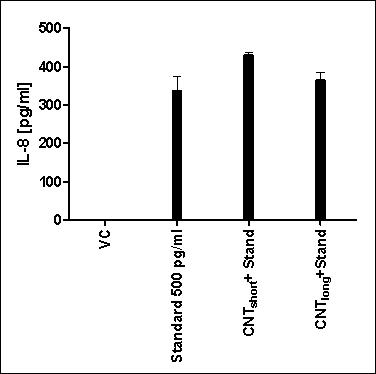


Supplementary Figure 2 Effect of CNT on levels of IL-8

Reference List

1. Murphy FA, Poland CA, Duffin R, Al Jamal KT, Ali-Boucetta H, Nunes A *et al*.: **Length-dependent retention of carbon nanotubes in the pleural space of mice initiates sustained inflammation and progressive fibrosis on the parietal pleura.** *Am J Pathol* 2011, **178:** 2587-2600.
